# Academic Output of Anesthesiology Departments in Canada From 2014 to 2023: A Bibliometric Analysis Study

**DOI:** 10.7759/cureus.82452

**Published:** 2025-04-17

**Authors:** Ekambir Saran, Connor T Brenna, Amrit Brar, Jiwon Lee, Daisy Thomas, Ella Huszti, Shiven Sharma, Karim S Ladha

**Affiliations:** 1 Temerty Faculty of Medicine, University of Toronto, Toronto, CAN; 2 Department of Physiology, University of Toronto, Toronto, CAN; 3 Department of Anesthesiology and Pain Medicine, University of Toronto, Toronto, CAN; 4 Institute of Health Policy, Management, and Evaluation, University of Toronto, Toronto, CAN; 5 Department of Otolaryngology, Icahn School of Medicine at Mount Sinai, New York, USA; 6 Department of Anesthesia and Pain Management, University Health Network, Toronto, CAN

**Keywords:** academic anesthesia, anesthesia research, bibliometric analysis, covid-19, publication trends

## Abstract

Anesthesia research is essential for advancing clinical practice and patient care. The purpose of this study was to analyze research productivity in Canadian anesthesiology departments from 2014 to 2023, focusing on trends in publication volume, methodology, and the impact of the COVID-19 pandemic. A bibliometric analysis was conducted following a pre-registered protocol to identify articles in the PubMed database, which were published between 2014 and 2023 (inclusive) with corresponding authors from Canadian anesthesiology departments. Data extracted for each article included the year of publication, journal, and study design. Descriptive statistics and Pearson correlation coefficient were used to compare trends, while annual publication rates were assessed with linear regression. An interaction term captured differences between pre-pandemic (2014-2020) and post-pandemic (2021-2023) periods. A total of 3,490 articles met the inclusion criteria. From 2014 to 2020 (pre-pandemic period), publication volume increased significantly by 28.7 studies/year (95% CI: 19.2-38.2, p < 0.001). In contrast, 2021-2023 (post-pandemic period) showed a non-significant decline of 13.0 studies/year (95% CI: -48.6-22.6, p = 0.405). Pre-pandemic trends showed significant growth in reviews, case-control/cohort studies, and surveys, while publication rates declined across most categories after 2020. Our findings illustrate an increase in research productivity among Canadian anesthesiology departments from 2014 to 2020, followed by a plateau in publication volume after the onset of the COVID-19 pandemic. This stagnation highlights a critical area for future exploration, including examining how pandemic-related factors, such as shifts in clinical priorities, resource allocation, and adoption of telemedicine in pre-operative clinics, have influenced research productivity. As the field of anesthesiology adapts to post-pandemic realities, ongoing bibliometric studies will be essential to monitor these trends and guide the trajectory of Canadian anesthesia research amid emerging clinical challenges and evolving academic priorities.

## Introduction and background

Anesthesia research plays a vital role in advancing the specialty by enhancing clinical practices and improving patient care. In Canada, academic institutions serve as the backbone of this research, driving progress through a wide range of studies, from preclinical science to clinical trials. Understanding trends in research output is crucial, as they not only reflect the vitality of the specialty but also guide its future directions.

Previous analyses have highlighted a concerning trend across Canadian anesthesia departments, revealing a decline in randomized controlled trials (RCTs) and stagnation in overall research activity during the period from 2000 to 2004 [1]. Although a follow-up study indicated that the decline in RCTs did not persist, the number of new annual RCTs fluctuated significantly, with only a minimal increase observed by 2013 [2]. Compounding these historical challenges, the COVID-19 pandemic introduced further obstacles, including the reallocation of resources and a temporary suspension of in-person appointments. During a lockdown that took place in Canada (and many other countries), elective surgeries were largely postponed, resulting in a significant halt in trial recruitment [3]. This raises the critical question of how anesthesia departments have responded to and navigated new challenges during and after the pandemic with respect to research activities.

To address these gaps in knowledge, our study aimed to analyze the research output of Canadian anesthesia departments over the past decade (2014-2023) and evaluate the impact of the COVID-19 pandemic on research productivity. We measured research productivity as the number of articles published in peer-reviewed journals [4].

## Review

Methodology

A bibliometric study was conducted to examine publications authored in Canadian anesthesiology departments over the past 10 years (2014-2023), following a protocol preregistered through the Open Science Framework (https://osf.io/5tykc) on 08 July 2024. The complete number of papers published over this time frame was obtained through a search in the PubMed database performed on 22 June 2024. The methodology for this study was adapted from a previous paper by Tsui et al. [1] to ensure comparability with historic trends in Canadian anesthesia research productivity. Research ethics board approval was not required as this study used only data available in the public domain.

Search terms were limited to the address of the corresponding author "[ad]", date published ‘‘[dp]", and publication type "[pt]", combined with the Boolean operators AND, OR, or NOT. The search parameters used included "anesthesia [ad] OR anaethesia [ad] OR anesthesiology [ad] OR anaesthesiology [ad] AND (Canada [ad] OR British Columbia [ad] OR BC [ad] OR Alberta [ad] OR Alta [ad] OR Saskatchewan [ad] OR Sask [ad] OR Manitoba [ad] OR Ontario [ad] OR Ont [ad] OR Quebec [ad] OR Que [ad] OR Newfoundland [ad] OR Nfld [ad] OR Nova Scotia [ad] OR NS [ad]) NOT letter [pt] NOT comment [pt]." The "publication date" was limited from 01 January 2014 to 31 December 2023.

**Figure 1:**
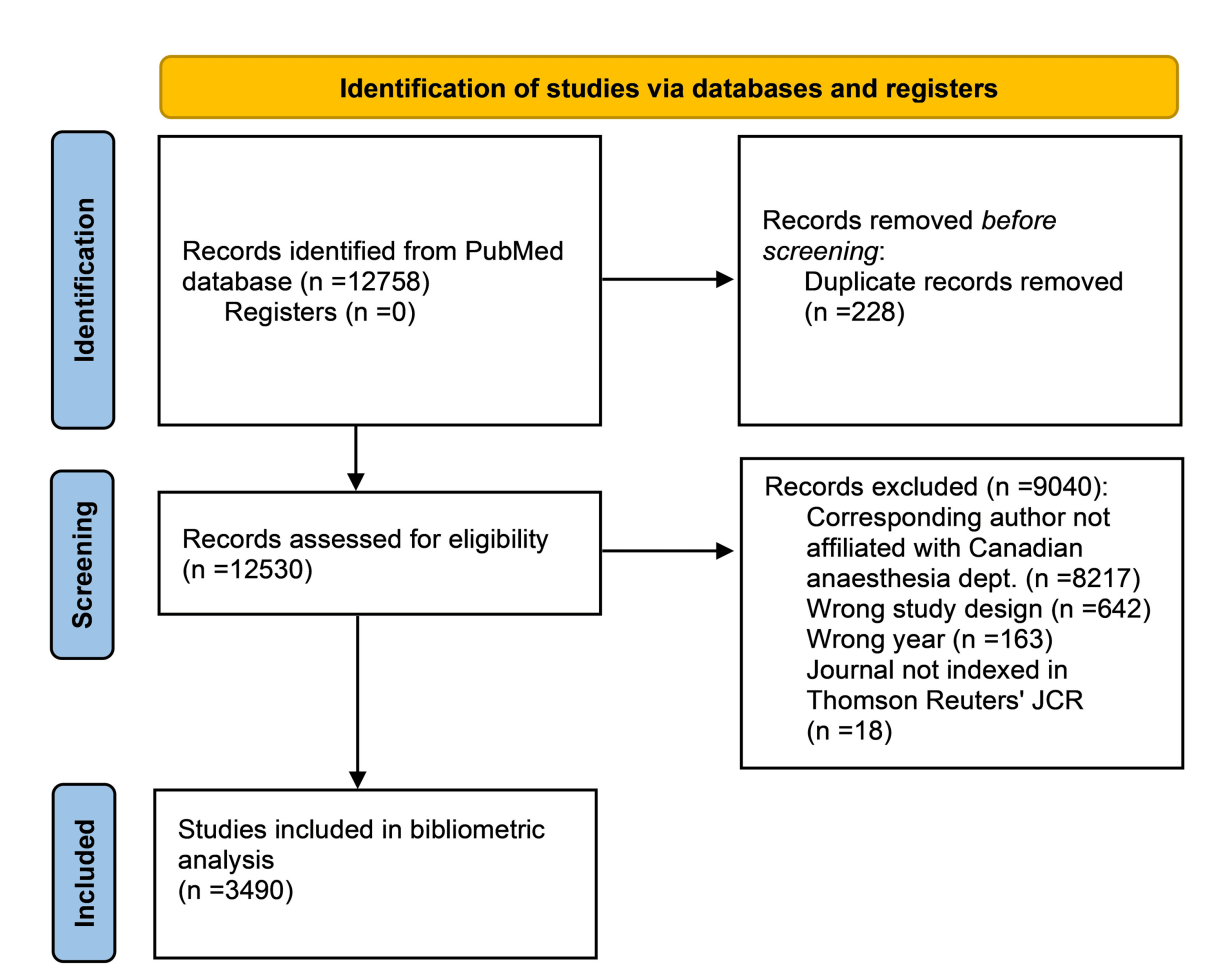
Flow diagram of the study selection.

Article titles and abstracts were screened for relevance using the Covidence software. Articles were considered eligible for inclusion if they were published in a peer-reviewed journal indexed in Thomson Reuters' Journal Citation Reports (JCR) with a corresponding author affiliated with a Canadian anesthesiology department between 2014 and 2023 (inclusive). Non-research publications, such as editorials, commentaries, book series, and letters that did not present original research, were excluded. No exclusions were made based on the language of publication. The study selection process is shown in the flow diagram constructed according to the Preferred Reporting Items for Systematic Reviews and Meta-Analyses (PRISMA) guidelines (Figure 1).

Extracted data included the journal in which the article was published, the year of publication, and the methodology. Included papers were classified into study designs based on an adaptation of the Cochrane Collaboration Glossary. Specifically, the research designs included animal studies/basic science research, case report/case series, case control/cohort, clinical trial/RCT, educational, review/systematic review/meta-analysis, survey, and other studies. As this report sought to understand trends in academic publication, rather than evaluate the quality of individual articles, we did not perform a risk of bias assessment.

To systematically categorize the data, articles were grouped by study design to identify prevalent research methodologies and by journal to pinpoint the leading outlets for anesthesiology research. Additionally, the data were further categorized by year, allowing for the analysis of trends in publication volume for each study design over time.

Descriptive statistics were used to compare the number of publications and study types published by authors affiliated with Canadian anesthesiology departments over the 10-year period (2014-2023). Linear regression was also used to determine the trend of the number of publications across all Canadian anesthesiology departments during this time period, using the year of publication as the independent variable and the number of publications as the dependent variable. In order to account for the potential shift in trend associated with the COVID-19 pandemic, an interaction term between publication year and a post-pandemic indicator was used. We defined 2014-2020 as the pre-pandemic period and 2021-2023 as the post-pandemic period to capture the natural temporal distinction before and after the disruptions caused by the COVID-19 pandemic, declared by the World Health Organization in March 2020. Furthermore, a pre-pandemic and post-pandemic Pearson correlation coefficient was calculated for each study design, and the statistical significance of the correlation was evaluated using a two-tailed t-test with five and one degrees of freedom for the two time periods, respectively. A coefficient of 0.7 or greater was considered to suggest a strong relationship between variables [5], and a p-value of <0.05 was used for statistical significance. All data were analyzed and presented using Prism 10.3.1 (Graphpad Software, Boston, MA) and Stata 18.0 (STATA Corp, College Station, TX).

Results

**Figure 2:**
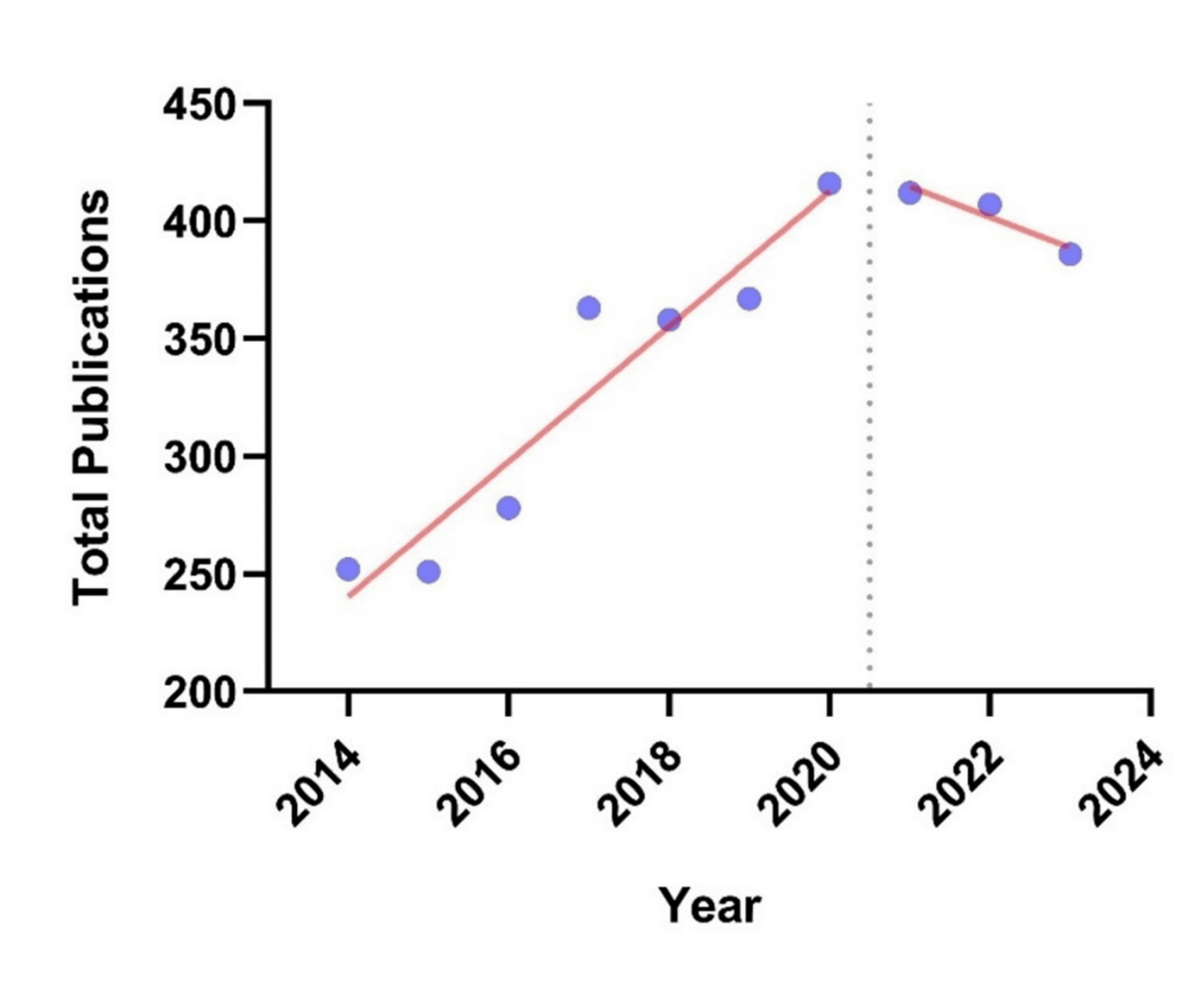
Trends in the annual publication volume of Canadian anesthesiology departments from 2014 to 2023. Total number of papers published by corresponding authors from Canadian anesthesiology departments, extracted from PubMed, between 2014 and 2020 (pre-pandemic) and 2021 and 2023 (post-pandemic). Each dot represents the annual publication volume, while the trendlines depict the linear regression model for each period.

The search strategy yielded 12,530 published studies, of which 3,490 (27.9%) met the inclusion criteria. Publication volume peaked in 2020 (416 articles) but declined steadily thereafter, resulting in a net 7% drop from 2021 to 2023. From 2014 to 2020, linear regression showed a significant annual increase in Canadian anesthesia publications, with a slope of 28.7 studies/year (95% CI: 19.2-38.2, p < 0.001), averaging a 11% yearly growth (Figure 2). Linear regression from 2021 to 2023 estimated a decline of 13.0 studies/year (95% CI: -48.6-22.6, p = 0.405).

**Figure 3:**
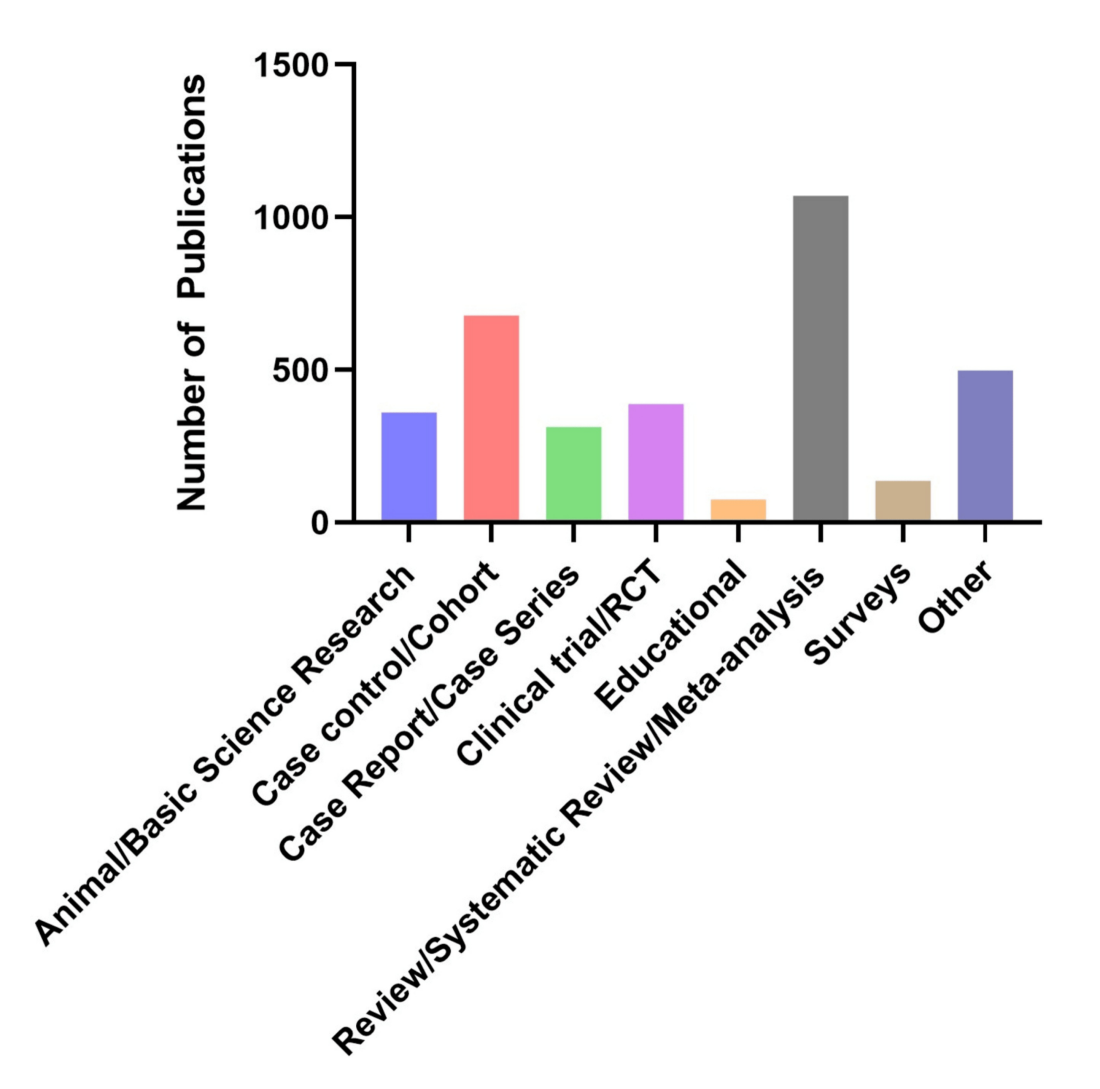
Types of anesthesia papers published by Canadian universities from 2014 to 2023.

The top three most frequently published, discrete study design categories over the 10-year period were reviews/systematic reviews/meta-analyses (1,069), case control/cohort studies (678), and clinical trials/RCTs (387), though the group of methodologies not fitting into these categories and classified as “other” was also large (498) (Figure 3). Study design categories with significant increases in frequency over 2014-2020 included reviews/systematic reviews/meta-analyses (r = 0.93, p ≤ 0.01), case control/cohort studies (r = 0.97, p ≤ 0.001), surveys (r = 0.93, p ≤ 0.01), and “other” studies (r = 0.98, p ≤ 0.001) (Table 1). Study designs with a negative publication trend over this time frame included clinical trials/RCTs (r = -0.38, p = 0.40), and educational studies (r = -0.15, p = 0.75). From 2021 to 2023, the rate of publications decreased across nearly all study designs compared to 2014-020, as reflected by a reduced Pearson correlation coefficient. The only exceptions were clinical trials/RCTs and educational studies.

**Table 1:** Study designs published by Canadian universities by year. * = p ≤ 0.05, ** = p ≤ 0.01, *** = p ≤ 0.001 RCT: Randomized controlled trials

Study Type	2014	2015	2016	2017	2018	2019	2020	2021	2022	2023	Pearson correlation coefficient (r) pre-pandemic (2014–2020)	Pearson correlation coefficient (r) post-pandemic (2021–2023)
Animal/Basic Science	37	32	34	33	35	39	33	48	38	31	0.09	-0.99
Case Report/Case Series	34	22	25	46	31	31	39	30	27	26	0.37	-0.96
Case Control/Cohort	39	39	51	67	75	75	84	76	103	69	0.97***	-0.19
Clinical Trial/RCT	41	40	39	47	39	41	34	36	34	36	-0.38	0
Educational	3	8	11	12	5	3	6	9	10	9	-0.15	0
Review/Systematic Review/Meta-Analysis	75	70	79	111	117	110	134	128	112	133	0.93**	0.23
Survey	5	11	7	12	14	18	23	19	15	13	0.93**	-0.98
Other	21	31	34	40	44	51	67	68	71	71	0.97***	0.87

The greatest number of articles was published in the Canadian Journal of Anesthesia (435 papers, 12.4%), followed by Anesthesia & Analgesia (168 papers, 4.8%), and Journal of Cardiothoracic and Vascular Anesthesia (112 papers, 3.2%) (Table 2).

**Table 2:** Top 10 journals with most Canadian anesthesia papers published from 2014 t 2023.

Journal	Number of papers (%)
Canadian Journal of Anesthesia	435 (12.4)
Anesthesia & Analgesia	168 (4.8)
Journal of Cardiothoracic and Vascular Anesthesia	112 (3.2)
Regional Anesthesia & Pain Medicine	95 (2.7)
Anesthesiology	77 (2.2)
British Journal of Anaesthesia	68 (1.9)
Pediatric Anesthesia	58 (1.7)
PLOS One	56 (1.6)
BMJ Open	54 (1.5)
Anaesthesia	49 (1.4)

Discussion

Academic Trends From 2014 to 2023

Our findings underscore a positive overall trend in anesthesia research output from Canadian institutions during the pre-pandemic period of 2014-2020, with an average increase in research output of 11% per year based on our linear regression model. The increase in research output culminated with a peak in 2020. During the post-pandemic period of 2021-2023, we observed a plateau in research output and a net 7% decrease in overall publication volume. Although this finding is associational, it suggests that the COVID-19 pandemic may have disrupted research activities in Canadian anesthesiology departments.

Comparative Insights and Historical Context

Our findings differ from earlier work by Tsui et al. [1], which reported that Canadian anesthesia publications remained relatively stable from 2000 to 2004. In contrast, we observed steady growth in research output over 2014-2020, reflecting the strengthening academic productivity of anesthesia departments across the country prior to the pandemic. This trend aligns with global anesthesia research, which has more than doubled from 3,302 annual articles in 1996 to over 7,000 in 2021 [6]. Similar growth has also occurred in other specialties, including orthopedic surgery, ophthalmology, and vascular surgery [7-9]. Additionally, the COVID-19 pandemic-induced declines in publication volume were not unique to anesthesia; a similar decrease in publication volume has been reported in other fields, such as a 5.69% decrease in orthopedic surgery [10], underscoring the pandemic's widespread impact on medical research.

Factors Influencing Research Productivity

Several factors could potentially have contributed to the overall increase in Canadian anesthesia research from 2014 to 2020. First, the growing anesthesia workforce, which expanded 1.8-fold between 1996 and 2018 [11], may have fostered a more vibrant research environment, potentially contributing to the increase in research productivity. Additionally, the competitive job market has driven Canadian anesthesia residents to seek further training to improve their employability, especially for academic hospital positions, perhaps leading to increased fellowship training and research activities in the field [12]. Notably, a significant surge in the publication of case control/cohort studies (r = 0.97, p ≤ 0.001), review articles (r = 0.93, p ≤ 0.01), and surveys (r = 0.93, p ≤ 0.01) over 2014-2020 contributed to the overall research growth across the field. In contrast, although not statistically significant, there was a declining trend in clinical trials/RCTs (r = -0.38, p = 0.40) over this time period, which may be attributed to challenges such as funding constraints, regulatory hurdles, and recruitment difficulties associated with conducting large-scale trials, compared to other types of research. Despite these challenges, RCTs still comprise about 9% of Canadian anesthesia publications in the decade of 2014-2023, which is comparable to the bibliometrics of other specialties such as ophthalmology, dermatology, general medicine, and general surgery [13,14].

Impact of COVID-19 on Research Productivity From 2021 to 2023

The COVID-19 pandemic brought unprecedented challenges to academic anesthesiology, disrupting an otherwise upward trajectory in research output. This may be attributable to several factors. Firstly, clinicians faced increased clinical responsibilities, limiting their academic time for research [15]. Furthermore, funding was largely redirected toward COVID-19 studies [16] and the pandemic exacerbated mental health challenges and burnout among anesthesiologists, which have persisted post-pandemic [17,18], potentially contributing to a sustained impact on research productivity within the field.

Globally, the pandemic significantly impeded clinical trial recruitment and development due to public safety measures, including lockdowns and service closures [19-21]. Interestingly, we did not observe a major decline in clinical trial/RCT publications across Canadian anesthesiology departments during the pandemic, as the number of these publications oscillated between 34 and 36 annually from 2021 to 2023. This could be attributable to several factors. Firstly, while trial recruitment was slowing down, we hypothesize that researchers may have been able to pivot towards early completion of ongoing trials, as well as completing the analysis and publication of trials, which preceded the pandemic. In fact, studies report that clinical trials often take long periods of time to publish after completion, as fewer than half of NIH-funded trials are published in a peer-reviewed biomedical journal indexed by Medline within 30 months of completion, with a median time to publication of approximately two years [22]. This lag suggests that much of the clinical trial/RCT output during the pandemic may reflect work completed before disruptions to trial recruitment occurred. If this hypothesis is true, it can be anticipated that a lapse in RCTs will follow the present dataset after some lag time. Furthermore, the number of clinical trials/RCTs published annually in Canadian anesthesiology departments is relatively low, meaning that minor fluctuations in publication volume may reflect natural variability rather than a significant pandemic-driven effect. Collectively, these factors may have contributed to the observed stability in RCT publications in Canadian anesthesiology research throughout the pandemic.

Limitations

Our study has several limitations that warrant consideration. First, we chose to evaluate Canadian anesthesia publications stored in the PubMed database. While MEDLINE, the primary database within PubMed, is a widely used resource, this choice may have led to the exclusion of some relevant publications exclusively indexed in other databases, potentially underrepresenting the overall research output of Canadian anesthesiology departments. Additionally, in assigning publications to Canadian universities, we based our analysis solely on the affiliation of the corresponding author, an approach that potentially overlooks the contributions of co-authors affiliated with Canadian anesthesiology departments on reports arising from international or inter-disciplinary collaborations. The classification of studies into specific design categories was based on title and abstract screening, which may have introduced misclassification bias, particularly for studies with ambiguous descriptions. For example, if individual study methodologies were ambiguous with respect to elements such as randomization, it was challenging to distinguish RCTs from prospective cohort studies. Finally, in the comparison of pre- and post-pandemic publication trends, conclusions were drawn from only three years of post-pandemic data, which is a relatively small sample size.

## Conclusions

This paper provides a comprehensive analysis of Canadian anesthesia research trends over the past decade to assess the evolution of the discipline. Our findings revealed an increase in research productivity from 2014 to 2020, followed by a plateau in publication volume correlating with the onset of the COVID-19 pandemic. In our dataset, these changes in publication trends were temporally associated with the COVID-19 pandemic; however, larger studies would be required to determine whether other factors could account for our observations. Nonetheless, this stagnation highlights a critical area for future exploration, including examining how pandemic-related factors such as shifts in clinical priorities, resource allocation, and the adoption of telemedicine in pre-operative clinics may have influenced research productivity. Another future direction arising from this work is to better understand the role that research support systems may play in incentivizing academic productivity, for example, whether factors such as protected research time and access to digital platforms can effectively bolster research outputs. As the field of anesthesiology adapts to post-pandemic realities, ongoing bibliometric studies will be essential to monitor these trends and guide the trajectory of Canadian anesthesia research in the context of emerging clinical challenges and evolving academic priorities.
